# Mechanical strain induces *ex vivo* expansion of periosteum

**DOI:** 10.1371/journal.pone.0279519

**Published:** 2022-12-30

**Authors:** Mary M. Walker, Molly E. Baumann, John H. Alexander, Britani N. Blackstone, Christopher B. Morgan, Thomas J. Scharschmidt, Heather M. Powell

**Affiliations:** 1 University Laboratory Animal Resources, The Ohio State University, Columbus, Ohio, United States of America; 2 Department of Biomedical Engineering, The Ohio State University, Columbus, Ohio, United States of America; 3 Department of Orthopaedics, Wexner Medical Center, The Ohio State University, Columbus, Ohio, United States of America; 4 Department of Materials Science and Engineering, The Ohio State University, Columbus, Ohio, United States of America; 5 Shriners Children’s Ohio, Dayton, Ohio, United States of America; University of South Carolina, UNITED STATES

## Abstract

Segmental bone defects present complex clinical challenges. Nonunion, malunion, and infection are common sequalae of autogenous bone grafts, allografts, and synthetic bone implants due to poor incorporation with the patient’s bone. The current project explores the osteogenic properties of periosteum to facilitate graft incorporation. As tissue area is a natural limitation of autografting, mechanical strain was implemented to expand the periosteum. Freshly harvested, porcine periosteum was strained at 5 and 10% per day for 10 days with non-strained and free-floating samples serving as controls. Total tissue size, viability and histologic examination revealed that strain increased area to a maximum of 1.6-fold in the 10% daily strain. No change in tissue anatomy or viability via MTT or Ki67 staining and quantification was observed among groups. The osteogenic potential of the mechanical expanded periosteum was then examined *in vivo*. Human cancellous allografts were wrapped with 10% per day strained, fresh, free-floating, or no porcine periosteum and implanted subcutaneously into female, athymic mice. Tissue was collected at 8- and 16-weeks. Gene expression analysis revealed a significant increase in alkaline phosphatase and osteocalcin in the fresh periosteum group at 8-weeks post implantation compared to all other groups. Values among all groups were similar at week 16. Additionally, histological assessment with H&E and Masson-Goldner Trichrome staining showed that all periosteal groups outperformed the non-periosteal allograft, with fresh periosteum demonstrating the highest levels of new tissue mineralization at the periosteum-bone interface. Overall, mechanical expansion of the periosteum can provide increased area for segmental healing via autograft strategies, though further studies are needed to explore culture methodology to optimize osteogenic potential.

## Introduction

Segmental bone loss secondary to infection, trauma, osteolysis, and tumor resection remains a challenging problem in orthopaedics and is associated with poor outcomes despite the large number of autogenous bone graft, allograft, synthetic bone substitutes that exist on the market [1, 2]. The management of large (>5 cm) intercalary defects are even more complicated as the use of autologous cancellous bone grafting strategies may not provide sufficient tissue to achieve union. These cases often require staged and time-consuming procedures such as the induced-membrane technique developed by Masquelet [3, 4] or distraction osteogenesis (DO) pioneered by Ilizarov [5–8]. In addition, other options including autogenous vascularized fibula grafts [9], intercalary endoprostheses [10], and structural allografts [11–15] are associated with unique complications that limit patient outcomes and lead to re-operation or failure of limb-salvage.

Bone allografts, either alone or in conjunction with osteogenic cells are commonly employed to address segmental bone defects. A myriad of different harvesting techniques, sites, and delivery systems have been developed, some of which are in clinical use at this time [16–18]. A common problem with these strategies is poor incorporation of the allograft with the host bone. Notably, one study found that after 5 years of implantation, on average, only 20% of most allografts are incorporated with the host bone [19]. A follow-up study by Enneking and Campanacci demonstrated similar findings with primarily appositional osteogenesis and limited graft incorporation beyond the allograft-host bone junction [20]. These retrieval studies highlight the key limitation of structural allografts: incorporation of large segmental allografts is, at best, incomplete and, in worst-case scenarios, completely lacking.

One potential strategy to improve outcomes is to exploit the osteogenic properties of periosteum to facilitate graft incorporation. Periosteum and the periosteal-derived mesenchymal stem cells it harbors play a crucial role in osteogenesis as well as fracture healing [21–24]. Furthermore, animal models have demonstrated improved rates of healing in critical-sized bone defects when autogenous periosteal grafts are combined with allografts [25–29]. Similar results have been observed with the utilization of autogenous medial femoral condyle periosteal flaps to facilitate union at the fracture or allograft-host junction non-unions [30–33].

Unfortunately, due to the limited size of these autogenous periosteal grafts, the prospect of wrapping an entire allograft or scaffold to achieve complete native bone incorporation is unfeasible at this time. One strategy under investigation to address this deficiency is to create a biomimetic tissue-engineered periosteum through the utilization of various biologic or synthetic scaffolds and subsequent seeding with mesenchymal stem cells [34]. This has demonstrated some early success in animal models [35–37]; however, it continues to underperform compared to autogenous bone grafting. *In vitro* periosteal expansion of established sources of autogenous periosteum is an intriguing alternative to biomimetic tissue engineering techniques. The concept of periosteal distraction has its origins in Ilizarov’s original work on the “Law of Tension-Stress,” which states that “tissues subjected to slow, steady traction become metabolically activated, a phenomenon characterized by the stimulation of both proliferation and biosynthetic cellular functions” [6, 7]. His pioneering work demonstrated that, during DO, the periosteum proliferates as well in response to strain [38]. Tension-induced periosteal proliferation is observed in maxillofacial and plastic surgery literature, where periosteal distraction is utilized to stimulate osteogenesis with the secondary effect of inducing periosteal proliferation [39–41]. Based on these findings, our goal was to apply tension to induce rapid periosteal expansion *in vitro* and subsequently utilize the expanded periosteum to enhance bone regeneration in an implanted allograft.

## Materials and methods

### Harvesting periosteum and *ex vivo* expansion

**Fig 1:**
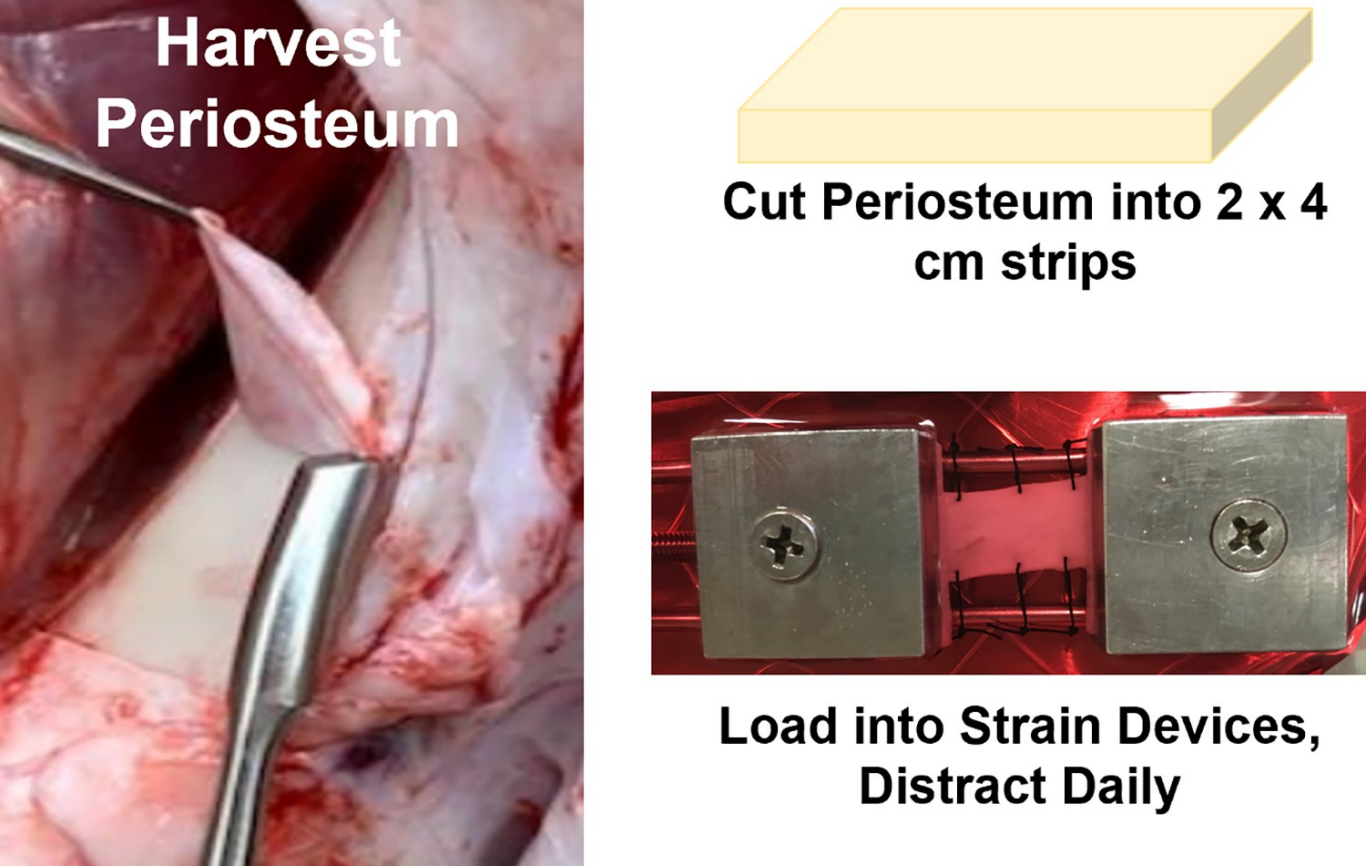
Harvesting of periosteum from the tibia of red Duroc pigs using a periosteal elevator. Periosteum was cut into 2 x 4 cm strips and loaded into custom made 316L stainless steel strain devices with long-axis edges constrained in the lateral direction using sutures.

Animals for this study were acquired on a protocol approved by The Ohio State University Institutional Laboratory Animal Care and Use Committee (IACUC, # 2014A00000072-R2). Animals were maintained at a surgical plane of anesthesia with isoflurane. While at a surgical plane of anesthesia, a lethal dose of saturated potassium chloride was administered IV and confirmation of euthanasia was made by absence of cardiac activity. Following euthanasia, strips of intact periosteum (2.0 x 4.0 cm) were harvested from the tibias of female Red Duroc pigs via subperiosteal dissection. HEPES-buffered saline (HBS) and 20% penicillin/streptomycin/fungizone (PSF) (Gibco, Waltham, MA) were used to disinfect the strips. Strips were maintained on M199 (Gibco) media supplemented with 5% fetal bovine serum (Gemini BioProducts, West Sacramento, CA) and 1% PSF. Samples from each pig were equally assigned to the different treatment groups: 10% strain, 5% strain, 0% strain (constrained control), and free-floating (unconstrained control). Strained samples were loaded into manual strain devices with a grip-to-grip distance of 2.0 cm with edges of the periosteum sutured to the outer guide rails of the strain device to maintain tissue width (Fig 1). The 10% group was manually strained twice a day (1 mm at each time) while the 5% strain group was manually strained once a day (1 mm/day). Strips were cultured with media changed every other day for 10 days. Photographs were taken each day and the area was assessed with computerized planimetry using ImageJ (NIH, Bethesda, MD).

Samples were embedded in OCT™, cryosectioned at 7 μm, and stained with hematoxylin and eosin (H&E). Additionally, slides were stained with DAPI and immunostained using Ki67 (Invitrogen, Waltham, MA) for quantitative analysis of cell proliferation (N = 6/group). H&E imaging was performed with brightfield microscopy (Phase Contrast-2, Nikon, Tokyo, Japan) and epifluorescent microscopy was performed with EVOS Auto 2 (Invitrogen). Quantification of total cells using DAPI staining was implemented with the MaxEntropy threshold command in ImageJ, whereas Ki67-postive cells were manually counted. Average percent Ki67 positive cells per field of view ± standard deviation was reported for each group (N = 6/group).

### Cell metabolism

At the conclusion of the 10 days, cell viability was assessed using an MTT assay. Briefly, two 6-mm biopsies were collected from each construct, placed individually in wells in a 24-well plate and incubated for 3 hours in a sterile 0.5mg/ml solution of thiazolyl blue tetrazolium bromide in phosphate-buffered saline. Following incubation, the solution was aspirated and 0.5 ml methoxy ethanol added. Plates were shielded from light and agitated on a rocker plate for 2 hours after which absorbance of the solution was read at 590 nm in a spectrophotometer (SpectraMax, Molecular Devices, San Jose, CA).

### *In vivo* assessment of bone deposition

All murine procedures were performed following a protocol approved by the Institutional Animal Care and Use Committee (#2018A00000072) at Ohio State University. Strips of periosteum were harvested from red Duroc pigs and disinfected as above. Periosteum from each pig was evenly assigned to each group (10% strain, free-floating, fresh, allograft only/control). The 5% strain group was not included in the murine studies as there was no statistically significant difference in total expansion, cell proliferation or cell metabolism between the 5% and 10% groups in the *in vitro* studies. Strained samples (N = 6) were loaded with a grip-to-grip distance of 3.0 cm. Samples were manually strained twice a day, 1.5 mm at a time, for 10% strain each day. Strained strips and free-floating (unconstrained) (N = 12) strips were cultured in M199 for 10 days with media exchanged 4 times per week. After 10 days of *in vitro* expansion, strained samples were removed from strain devices, cut in half and wrapped around cancellous bone allografts (5 x 5 x 15 mm, MTF Biologics, Edison, NJ) and sutured into place (Nylon 6–0, Ethicon, Somerville, NJ). Free-floating samples were wrapped around bone allografts using the same procedure. Fresh periosteum was collected as above on the day of surgery, immediately disinfected and wrapped around the bone allograft (N = 12). Cancellous bone allografts without periosteum were implanted as controls (N = 12).

Three days prior to surgery, Ibuprofen (100mg/5mL, Children’s Motrin Oral Suspension, Johnson & Johnson, Fort Washington, PA) was added to the drinking water at a calculated intake dosage of 40mg/kg/day based on normative consumption data of 5mL/day for a 25g mouse. The day prior to surgery, 1mg/kg buprenorphine SR (1.0 mg/mL stock, ZooPharm, Windsor, CO) was administered. Anesthesia was induced using 5% inhaled isoflurane and maintained at a surgical plane of anesthesia with 1–2% isoflurane. A toe pinch was performed to confirm anesthetic depth prior to manipulation. Constructs were then implanted into a subcutaneous pocket on female, immunodeficient mice (athymic nude-*FOXn1*^nu^; Envigo, Indianapolis, IN) weighing at least 22 g to ensure sufficient body size for two contralateral grafts. All mice within the cohort met this inclusion criteria. Samples were randomly assigned to mice with each mouse having a different bone graft on each side. The skin was prepared with three alternating scrubs of betadine (Fisher Scientific, Waltham, MA) and isopropyl alcohol (Covidien, Dublin, Ireland). While under anesthesia (1% inhaled isoflurane), a small incision was made in both flanks of the mouse in the dorsal-ventral direction and a subcutaneous pocket created using blunt forceps. The construct was inserted into the pocket and the incision closed with nylon sutures. Wounds were dressed with xeroform (Covidien), followed by Tegaderm™ (3M, St. Paul, MN) and Coban (3M). Animals and dressings were assessed daily with dressings and sutures removed 10 days post-implantation. For the duration of the study, mice were housed in groups with free access to food and water and supplemental enrichment including cotton neslets and enrichment huts. Mice were monitored daily when in dressings and three times per week post dressing removal. Mice were removed from study if any met early exclusion criteria; however, no mice experienced adverse events during this study.

### Histological analysis

At 16-weeks post-implantation, mice (N = 6 per group at each time point) were euthanized via CO_2_ inhalation with cervical dislocation to confirm euthanasia. Implants were removed and cut in half. One half was fixed in 70% ethanol and embedded in methylmethacrylate. Undecalcified blocks were cut at 10 μm and stained with Masson-Goldner Trichrome (Sigma-Aldrich, St. Louis, MO) to evaluate new bone deposition.

### Quantitative gene expression analysis

Specimens collected at 8- and 16-weeks post-implantation were snap-frozen and pulverized using a cryogenic tissue grinder (Spex Sample Prep, Metuchen, NJ). Total RNA was purified following the manufacturer’s instructions with the Norgen Animal Tissue RNA purification kit (Norgen Biotek Corp., Thorold, Ontario, Canada). RNA samples were treated with DNase I (Qiagen, Inc., Germantown, MD) prior to cDNA Synthesis with SuperScript VILO (ThermoFisher Scientific, Waltham, MA). Quantitative PCR (qPCR) was performed using porcine gene-specific primers for alkaline phosphatase (ALP), decorin, osteoprotegerin, and osteocalcin (RT^2^ qPCR Primer Assays; Qiagen, Inc.), RT^2^ SYBR® Green qPCR Mastermixes (Qiagen, Inc.) and the iCycler IQ system (Bio-Rad Laboratories, Inc., Hercules, CA). Samples were analyzed using technical triplicates in addition to biological replicates (N = 6). Expression levels were referenced to the glyceraldehyde 3-phosphate dehydrogenase (GAPDH) gene using the comparative 2-ΔΔCt method [42] to control for mRNA levels per cell.

### Statistics

Data were analyzed using SigmaPlot 13.0 (Systat Software Inc., San Jose, CA). To evaluate differences between scaffold types, One Way analysis of variance (ANOVA) with a Tukey *post-hoc* test was used. Statistical significance was reported for p values less than 0.05.

## Results

### *In vitro* strain increases amount of total periosteum available

**Fig 2:**
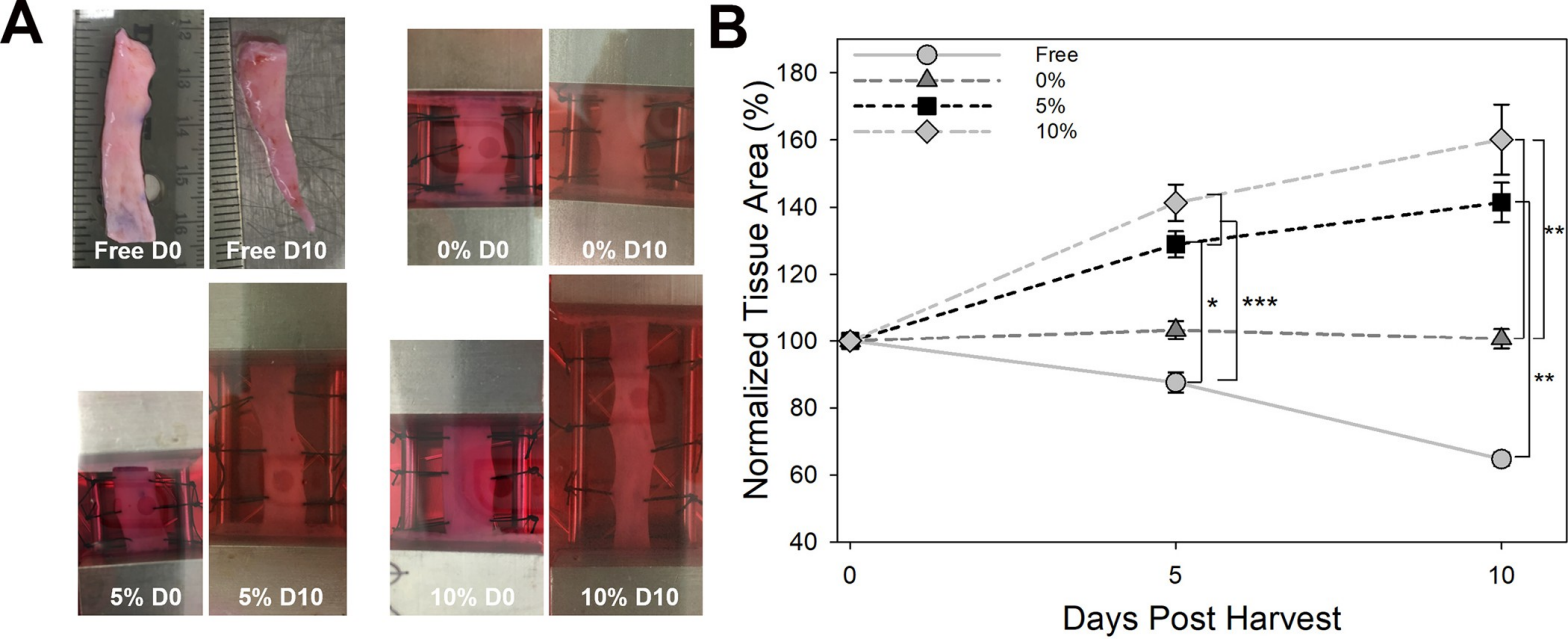
*Ex vivo* expansion of periosteal tissue is not linear with total applied strain. A) Photographs of periosteum at day 0 and after 10 days of culture. Note the decrease in periosteal strip width in the 10% group. B) Quantification of periosteal area normalized to day 0.

The strained periosteum remained intact throughout culture with contraction observed perpendicular to the principal direction of strain in the 5 and 10% groups (Fig 2A). In the unconstrained group, pieces of periosteum appeared to shrink with time (Fig 2A). Quantitative analysis of tissue area as a function of time revealed a significant change in size during the culture period (p < 0.001) with the free-floating group contracting to roughly 62% of the original area by day 10 (Fig 2B). Both the 5 and 10% strain groups were expanded to a greater area than the constrained group; however, there was no significant difference between groups (Fig 2B). The greatest expansion was observed in the 10% group with ~1.6-fold expansion at day 10.

### *In vitro* strain maintains tissue viability and anatomy

**Fig 3:**
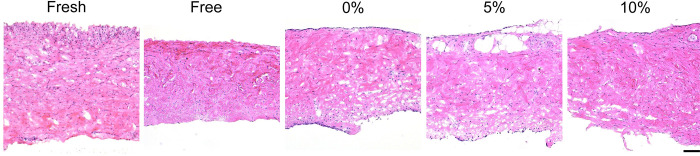
Anatomy of periosteal tissue prior to and following *in vitro* culture. H&E stained sections of fresh porcine periosteal tissue and periosteum after 10 days in culture. Scale bar = 100 μm.

**Fig 4:**
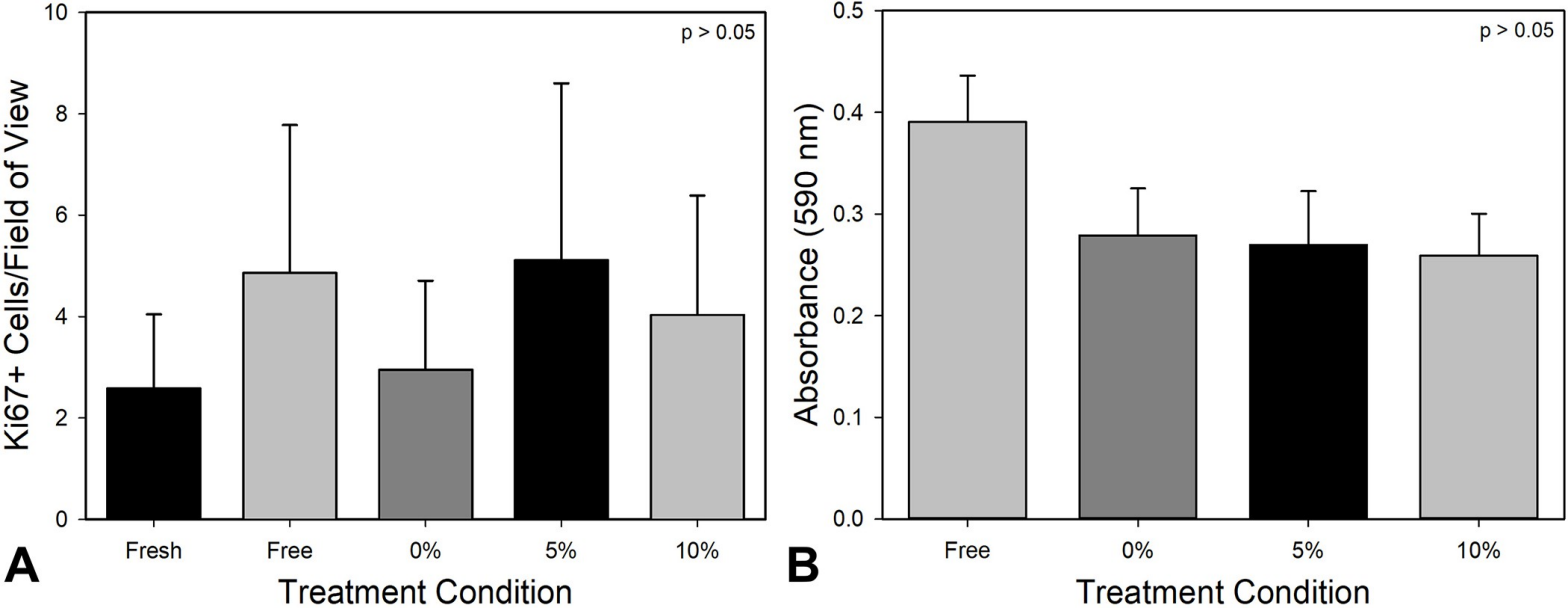
*Ex vivo* expansion does not significantly alter cell proliferation or metabolism. A) Quantification of Ki67^+^ cells per field of view as a function of culture condition. B) MTT cellular metabolism assay performed on punch biopsies collected from periosteum on culture day 10.

Cellularity and tissue thickness was reduced following free-floating culture for 10 days; however, constrained and strained periosteum maintained tissue thickness (Fig 3). Immunostaining for DAPI and Ki67 showed that there was not a significant change in cell proliferation among fresh or cultured periosteum and that strain did not have a large effect on proliferation as measured on day 10 (Fig 4A). MTT analysis showed no significant difference in cellular metabolism as a function of strain (Fig 4B). The free-floating group, on average has a higher absorbance; however, this was not statistically significant. Additionally, a comparison of fresh versus cultured periosteum showed similar tissue anatomy in all groups.

### Presence of periosteum enhances allograft remodeling and mineralization

**Fig 5:**
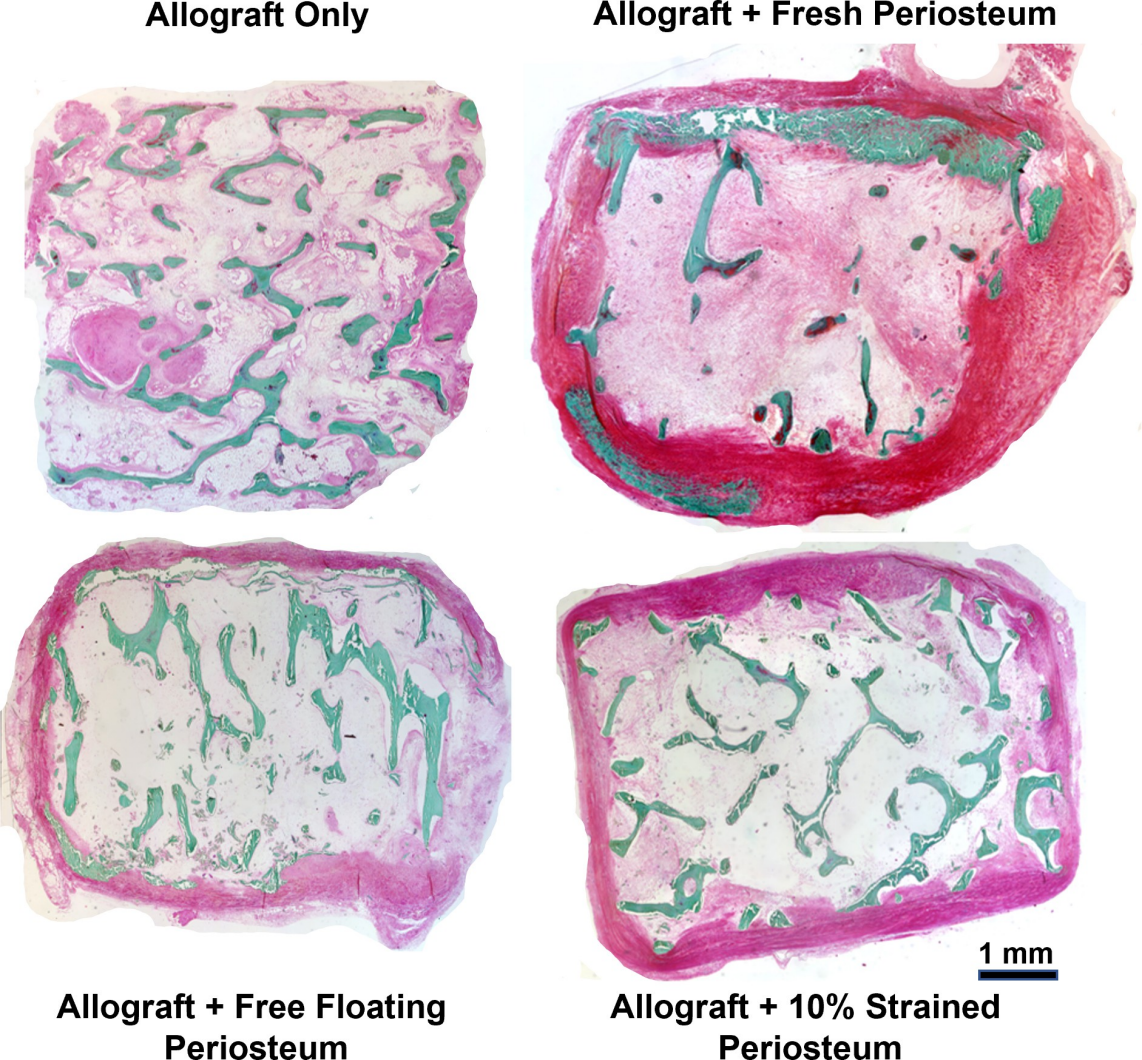
Mineralization in bone allograft following subcutaneous implantation. Masson-Goldner staining of undecalcified sections from allograft only, allograft wrapped with fresh periosteum and allograft wrapped with mechanically expanded periosteum 16 weeks post-implantation.

After 16 weeks *in vivo*, tissue infiltration was observed in all groups (Fig 5). In the allograft only group, the original struts of the cancellous grafts are clearly visible with soft tissue present within the trabeculae. In contrast, the allograft wrapped with fresh periosteum had little original allograft remaining with new mineralized tissue at the periphery of the allograft near the periosteum (Fig 5). A small amount of allograft remodeling was observed in the free-floating and 10% strained groups with lower amounts of new tissue infiltrate compared to the fresh periosteum and allograft alone groups.

**Fig 6:**
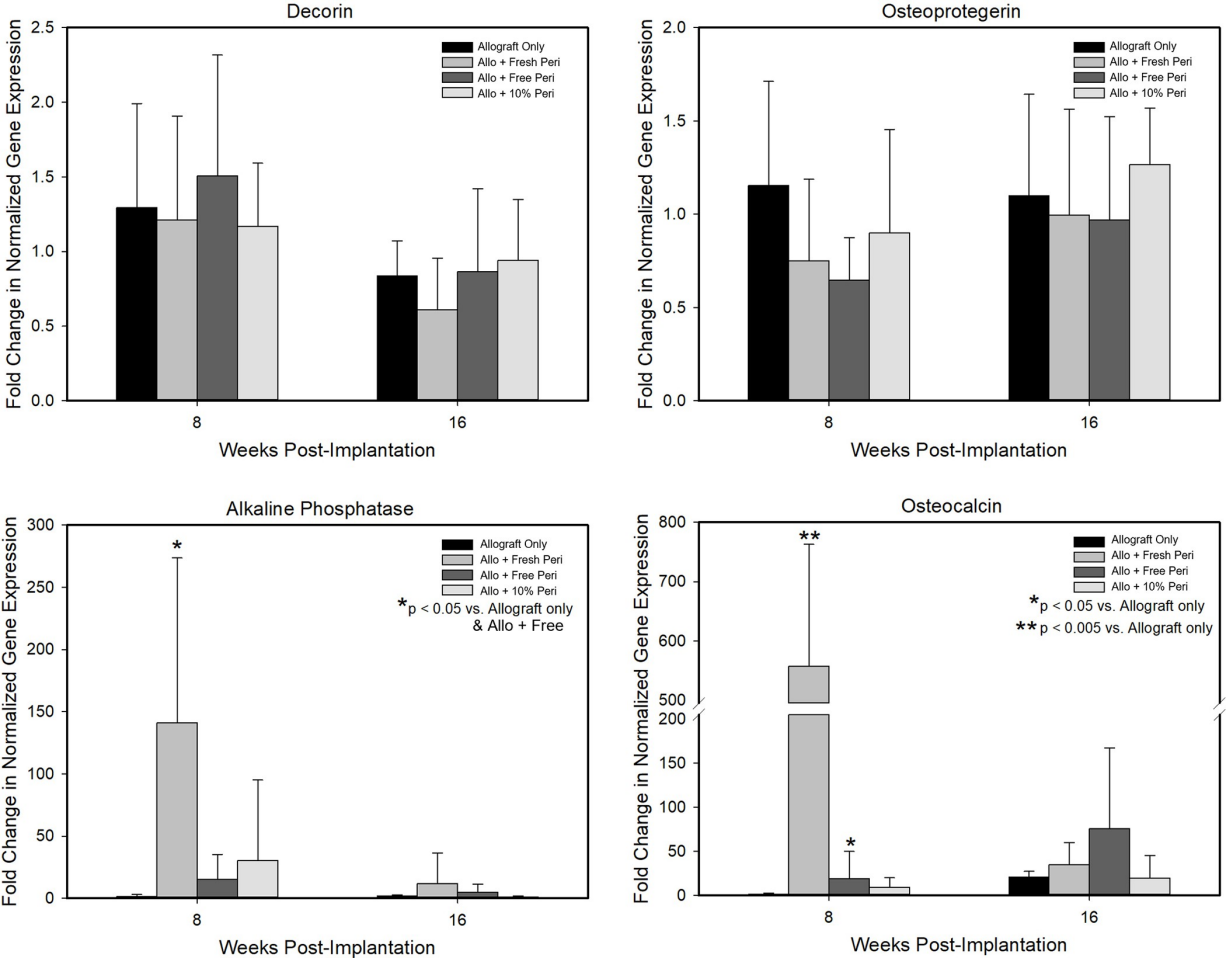
Expression of genes related to bone regeneration in allograft alone or wrapped with periosteum. Normalized gene expression for decorin, osteoprotegerin, alkaline phosphatase, and osteocalcin from samples collected at 8 and 16 weeks post-implantation.

No significant difference in decorin and osteoprotegerin gene expression was observed among the groups at 8 or 16 weeks and there were no changes in expression with time (Fig 6). Alkaline phosphatase expression was greatest in all groups at the week 8 timepoint with significantly greater expression in the allograft + fresh periosteum group. Expression of osteocalcin was also greatest in the allograft + fresh periosteum group at week 8 with expression reduced at the 16-week time point in this group. For all other groups expression of osteocalcin increased from week 8 to 16 (Fig 6).

## Discussion

In Ilizarov’s seminal study, the optimal rate of distraction osteogenesis as applied to limb lengthening was 1mm, implemented in 60 adjustments per day [6]. The current study utilized 5% (1mm/once a day) strain and 10% (1mm/twice a day) uniaxial strain with the edge of the periosteum sutured to strain device. Though it was anticipated that there would be a linear relationship between applied strain and total area of periosteum, contraction of the tissue in the y-direction resulted in no significant difference in amount of total periosteum available between strained groups. Distraction techniques must maintain a balance between proliferation enhancement and expansion beyond physiologic limit. Exceeding such limits can result in apoptosis, necrosis, low-density tissue, and contraction [43–46]. Contraction was observed perpendicular to the principal direction of strain in both the 5% and 10% samples, indicating that these parameters may have exceeded physiologic strain. Several studies support a saturation point of strain; thus, the use of a more sophisticated mechanical bioreactor may be beneficial [47–50].

There is a large body of evidence to support that mechanical strain enhances proliferation. Human and animal models have directly and indirectly established strain rate as a key driver of osteogenesis independent of strain magnitude [51–55]. Similar to physical increase in area, the effect of induced strain on cellular proliferation must achieve biomimetic balance. Previous tension-stress studies underscore the conclusion that rapid strain can lead to proliferation in different cellular populations; for example, Ilizarov’s found that 2.0 mm strain completed in 4 daily steps resulted in fibrous tissue deposition with no osteogenic activity in canine tibias [6]. In contrast, strain rates that are too slow may be outpaced by physiologic healing. The current study quantified *in vitro* proliferation based on Ki67^+^ populations within the periosteum. Though the current data suggest no difference in proliferation within the porcine periosteum, it is unclear which populations are proliferating. The Ki67^+^ populations were within the cambium layer; however, a lack of porcine specific antibodies to label osteogenic progenitors hindered efforts to examine which populations were proliferating in response to the mechanical stimuli. In future studies, gene-based analyses may be utilized to examine shifts in osteogenic potential of the periosteum before and after mechanical expansion *in vitro*.

Periosteum in the current study was harvested from cortical bone both for the ultimate clinical goal of maximizing donor tissue and to isolate it in culture for focused characterization of proliferation and gene expression. This introduces several disadvantages compared to *in vivo* expansion. First, the manual strain device applied a static force as opposed to incremental or cyclic strain that may better mimic the dynamic *in vivo* environment [47, 56, 57]. Cyclic strain experiments have resulted in higher cell density [58], higher MSC expression of bone morphogenic protein 2 (BMP2) [59], and increases in both periosteal and endocortical perimeter [60] as compared to static or no applied strain. Another disadvantage is that fresh tissue is optimal for maintenance in culture, but clinical applications of periosteal grafting often employ a period of latency between placement and distraction [6, 49, 61]. The current study’s 0% (constrained) samples may approximate tissue behavior in this latency period, but the same tissue was not subjected to subsequent strain. Lastly, osteogenic interplay between cortical bone and periosteum is well-established [40, 62–64]. Studies that compared periosteal distraction osteogenesis (PDO) with DO techniques found that DO outperformed PDO in area expansion [62] and osteogenic density [63]. The current culture technique may have disadvantaged expansion efforts by isolating the periosteum; tissue harvest of both cortical bone and the periosteum could enhance *in vitro* strain experimentation. Alternatively, culture media may be enriched with MSCs to approximate cortical bone perforation and contribution [63, 64].

As anticipated, *in vivo* implantation of periosteum with the allograft construct was associated with increased mineral deposition compared to allograft alone, demonstrating the beneficial coordination between periosteal and cortical tissue consistent with previous studies [3, 31, 65]. Mechanical strain has been shown previously to control MSC proliferation and differentiation with lower strain rates (3%) resulting in differentiation of MSCs into osteo-related cells while higher rates (10%) resulted in a smooth muscle-like phenotype [66]. Similar studies have shown that α-smooth muscle actin indicates quiescent skeletal stem cells in adult periosteal tissue [67]. In the current study, no change in cell metabolism was observed among the groups as a function of strain; however, significant differences in bone deposition and expression of osteogenic genes were observed. As fresh periosteum resulted in greater mineral deposition compared to strained samples, it is possible that MSCs and other osteogenic progenitors were reduced in number while the fibroblasts were maintained or proliferated during the culture period.

Gene expression results further supported the benefit of periosteum in bone regeneration *in vivo*. Whereas alkaline phosphatase and osteocalcin were not detected in allograft-only implants, these respectively early and late markers of bone formation were present in cancellous bone and periosteum constructs. The highest levels were detected in the fresh periosteal sample, which is surprising, given that ALP and osteocalcin have specifically been shown to increase as a result of biomechanical strain, similar to other osteogenic proteins [68]. This prior study, however, was performed entirely *in vivo*, whereas the strain component of the current investigation was *in vitro* prior to construct placement in the murine model. Ilizarov’s experiments similarly reported low levels of ALP at 1mm once daily rates, but high levels when an autodistractor expanded 1mm/day in 60 increments [6]. Interestingly, 2 mm distraction in 4 daily steps was lower than a 1mm distraction in 4 daily steps, reinforcing the strain saturation hypothesis.

## Conclusion

*Ex vivo* tension can be utilized to expand the quantity of periosteum; however, the magnitude of expansion is limited when applied using 1–2 strain events per day. Fresh and mechanically expanded periosteum significantly improved allograft remodeling and bone deposition *in vivo*; however, outcomes were significantly improved with fresh periosteum. Future methods should seek to optimize the rate of strain application and culture environment to expand the tissue while maintaining osteogenic cell populations.

## Supporting information

S1 Checklist (PDF)
